# Adenosine Receptors as Neuroinflammation Modulators: Role of A_1_ Agonists and A_2A_ Antagonists

**DOI:** 10.3390/cells9071739

**Published:** 2020-07-21

**Authors:** Aleix Martí Navia, Diego Dal Ben, Catia Lambertucci, Andrea Spinaci, Rosaria Volpini, Inês Marques-Morgado, Joana E. Coelho, Luísa V. Lopes, Gabriella Marucci, Michela Buccioni

**Affiliations:** 1 School of Pharmacy, Medicinal Chemistry Unit, University of Camerino, 62032 Camerino (MC), Italy; aleix.martinavia@unicam.it (A.M.N.); diego.dalben@unicam.it (D.D.B.); catia.lambertucci@unicam.it (C.L.); andrea.spinaci@unicam.it (A.S.); rosaria.volpini@unicam.it (R.V.); michela.buccioni@unicam.it (M.B.); 2 Instituto de Medicina Molecular, Faculdade de Medicina de Lisboa, Universidade de Lisboa, 1649-028 Lisbon, Portugal; anamorgado@medicina.ulisboa.pt (I.M.-M.); jfecoelho@medicina.ulisboa.pt (J.E.C.); lvlopes@medicina.ulisboa.pt (L.V.L.)

**Keywords:** A_1_AR agonist, A_2A_AR antagonist, neuroinflammation, cytokine, LPS, glia

## Abstract

The pathological condition of neuroinflammation is caused by the activation of the neuroimmune cells astrocytes and microglia. The autacoid adenosine seems to be an important neuromodulator in this condition. Its main receptors involved in the neuroinflammation modulation are A_1_AR and A_2A_AR. Evidence suggests that A_1_AR activation produces a neuroprotective effect and A_2A_ARs block prevents neuroinflammation. The aim of this work is to elucidate the effects of these receptors in neuroinflammation using the partial agonist 2′-dCCPA (2-chloro-N^6^-cyclopentyl-2′-deoxyadenosine) (C**1** K_i_A_1_AR = 550 nM, K_i_A_2A_AR = 24,800 nM, and K_i_A_3_AR = 5560 nM, α = 0.70, EC_50_A_1_AR = 832 nM) and the newly synthesized in house compound 8-chloro-9-ethyl-2-phenethoxyadenine (C**2** K_i_A_2A_AR = 0.75 nM; K_i_A_1_AR = 17 nM and K_i_A_3_AR = 227 nM, IC_50_A_2A_AR = 251 nM unpublished results). The experiments were performed in in vitro and in in vivo models of neuroinflammation. Results showed that C**1** was able to prevent the inflammatory effect induced by cytokine cocktail (TNF-α, IL-1β, and IFN-γ) while C**2** possess both anti-inflammatory and antioxidant properties, counteracting both neuroinflammation in mixed glial cells and in an animal model of neuroinflammation. In conclusion, C**2** is a potential candidate for neuroinflammation therapy.

## 1. Introduction

Neuroinflammation comprises a wide range of biological and cellular responses of the nervous system to injury, infection, and neurodegenerative diseases. This pathological condition is caused by the activation of the neuroimmune cells from the brain astrocytes and microglia. Deregulation of microglia and astrocytes, abnormal pro-inflammatory cytokines (CK) synthesis such as tumor necrosis factor-α (TNF-α), interleukin 1 beta (IL-1β), and interferon-γ (IFN-γ), and rise of reactive oxygen and nitrogen species are the most common aspects in neuroinflammation [1,2,3,4]. It has been demonstrated that neuroinflammation plays a pivotal role in many neurodegenerative diseases such as Alzheimer’s and Parkinson’s diseases, amyotrophic lateral sclerosis, Huntington disease, spinocerebellar ataxia, and multiple sclerosis [5,6].

In spite of these pathologies have different etiologies such as infections, genetic mutations, trauma, and protein aggregations, etc., the common neuronal damage, in the central nervous system (CNS), is associated with chronic activation of an innate immune response. In addition, in multiple sclerosis there is an infiltration of immune cells across the blood brain barrier (BBB) [7].

Microglia and astrocytes, the brain resident immune cells, in normal conditions develop several biological actions as protection or synthesis of anti-inflammatory molecules, but once activated they produce several aggressive agents like pro-inflammatory CK, TNF-α, IL-1β, and INF-γ, or reactive oxygen species [1]. The up-regulation of pro-inflammatory CK plays multiple roles in both neurodegeneration and neuroprotection [8]. Recently, the adenosine receptors (ARs) are emerging as an attractive therapeutic target for modulating brain injury in a variety of animal models of neurological disorders [9,10]. Adenosine is an endogenous nucleoside widely distributed throughout the body where it regulates many functions. ARs belong to G-protein coupled receptors and are divided into A_1_, A_2A_, A_2B_, and A_3_ receptors. In the brain was demonstrated a higher density of A_1_AR and A_2A_AR. Adenosine, in the CNS, acts as neuromodulator and as a homeostatic modulator. Its neuromodulator role, on excitatory glutamatergic synapses, depends on a balanced activation of inhibitory A_1_AR and excitatory A_2A_AR. In fact, A_1_AR activation modulates negatively the excitatory transmission, while A_2A_R activation promotes synaptic plasticity [11].

In physiological conditions, extracellular adenosine level is around nanomolar concentration. After brain injury the level is forcefully increased, even if many of adenosine effects protect neuronal integrity, it, in the same events, exacerbates neuronal injury by promoting inflammatory processes [12]. The main receptors involved in neuroinflammation modulation are A_1_AR and A_2A_AR. The A_1_AR is expressed in microglia and plays an important role on its activation [13]. In A_1_AR knockout mice there is an increase in neuroinflammation and microglia activity [14]. This finding suggests that the A_1_AR activation produces a neuroprotective effect in pathological conditions. Additionally, in physiological and/or pathological conditions, adenosine, by A_1_AR activation, reduced the astrocyte proliferation and induced the release of nerve growth factor (NGF) [15]. In summary, A_1_AR is a critical endogenous physiological regulator in neurons and it may be potential therapeutic target in neuroinflammation.

The A_2A_AR, in physiological conditions, is highly expressed in striatal neurons and less in glial cells and neurons outside the striatum. This receptor subtype is also expressed in the nucleus accumbens, caudate putamen, and olfactory tubercle [16]. It is worth noting that A_2A_AR expression in microglia increases as a result of brain insults leading to signal transductions that do not occur in cells with the receptors expressed at a normal level such as facilitating the release of cytokines [17]. On the other hand, A_2A_AR antagonists suppress microglia activation in in vitro and in vivo studies [18,19,20,21].

Since A_1_AR activation produces a neuroprotective effect and A_2A_ARs block neuroinflammation, in this work studies with an A_1_AR agonist and an A_2A_AR antagonist were performed in in vitro and in in vivo models of neuroinflammation.

**Figure 1:**
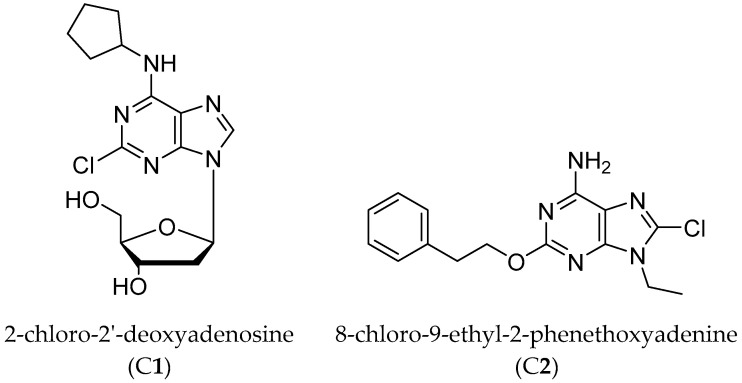
Compound **1** and **2** structures.

Hence, the A_1_AR partial agonist 2-chloro-N^6^-cyclopentyl-2′-deoxyadenosine (2′-dCCPA, C**1**) [22] and the A_2A_AR antagonist 8-chloro-9-ethyl-2-phenethoxyadenine (C**2**) [unpublished], which were synthesized in our laboratory, were utilized (Figure 1).

The 2′-dCCPA (C**1**) showed a submicromolar A_1_AR affinity in binding studies (K_i_A_1_AR = 550 nM, K_i_A_2A_AR = 24,800 nM, and K_i_A_3_AR = 5560 nM) performed in Chinese hamster ovary cells (CHO) stably transfected with the human ARs. Functional studies performed at *h*A_1_AR expressed in the same cells revealed, by evaluating the inhibition of adenyl cyclase activity induced by forskolin, that this compound is endowed with a partial agonist behavior (α = 0.70, EC_50_A_1_AR = 832 nM). This result was confirmed in an ex vivo experiment performed at mouse ileum contractility, where it presented to induce the 75% maximal reduction of contractility obtained with the full agonist 2-chloro-N^6^-cyclopentyladenosine (CCPA) [23].

The partial agonist behavior could be beneficial in the treatment of acute and chronic disease due to less side effects respect to the A_1_AR full agonist. In addition, C**1** showed to protect SH-SY5Y cells from oxygen-glucose deprivation (OGD) at different concentrations representing a possible alternative for the management of cerebral ischemia and thus also for the management of neuroinflammation [23].

C**2** belongs to a series of new 8-substituted 9-ethyl-2-phenethoxyadenines, synthesized with the aim to improve the affinity at the A_2A_AR of the 8-bromo-9-ethyl-2-phenethoxyadenine (K_i_A_2A_AR = 1.7 nM) [24]. C**2** resulted an A_2A_AR antagonist with a subnanomolar affinity and a good selectivity (K_i_A_2A_AR = 0.75 nM; K_i_A_1_AR = 17 nM and K_i_A_3_AR = 227 nM, IC_50_A_2A_AR = 251 nM unpublished results).

## 2. Experimental Procedures

### 2.1. Materials

Compounds **1** and **2** were synthesized in the School of Pharmacy of Camerino University (Italy), and dissolved in dimethylsulfoxide (DMSO) to prepare a 0.01 M stock solution, which was then diluted with water to the concentration required for the experiments. In all experiments, the maximum concentration of DMSO in wells did not exceed 0.5% and had no effect on cell viability. CCPA, ZM241385, SCH58261, DPCPX, and Hoechst 33258 were purchased from Sigma-Aldrich (Milan, Italy). All material concerning cell culture was purchased from EuroClone S.p.A. (Milan, Italy), Cytokines were purchased from S.I.A.L. S.r.l. (Rome, Italy), CellTiter 96^®^ Aqueous One Solution Cell Proliferation Assay and Griess Regent System were purchased from Promega (Milan, Italy). Antibodies of rabbit anti-adenosine receptor A_1_ (Bioss Company, bs-6649R) and rabbit anti-adenosine receptor A_2A_ (Bioss Company, bs-1456R) were bought from S.I.A.L. S.r.l. (Rome, Italy). 

### 2.2. In Vitro Assays

All animal experiments were carried out according to the principles and procedures outlined in the European Community Guidelines for Animal Care, DL 26/2014, application of the European Communities Council Directive, 2010/63/EU. 

#### 2.2.1. Mixed Glial Cell Culture

Newborn male Wistar rats were sacrificed and mixed glial cell cultures were obtained from the cerebral cortices. Cerebral cortices were dissected, and meninges were cleared away. Tissues were treated with trypsin solution (trypsin 0.05% (w/v) trypsin-EDTA 5 mM) for 25 min at 37 °C. The tissue was fully disaggregated by pipetting and the dissociated cells were seeded in a 6-well plate with Dulbecco’s modified Eagle’s/High glucose medium enriched with 100 U/mL penicillin, 100 µg/mL streptomycin and 10% FBS. Fresh medium was changed the next day and then every 3 days decreasing the quantity of FBS. All experiments were carried out after 7–9 days of plating [25].

#### 2.2.2. Primary Neuronal Cell Cultures

Neurons were obtained from hippocampus or cortex of embryos. Briefly, pregnant rat was scarified and the uterus removed. Embryos were taken out from the uterus and brains were obtained. After removing the meninges, hippocampus and cortex were dissected and homogenated with tweezers. The result was incubated with trypsin for 15 min at 37 °C and then centrifuged 2 min at 1000 rpm. The pellet was resuspended with 15 mL of HBSS/0.37% Glucose/30% FBS and centrifuged as before for three times. Finally, the pellet was resuspended with 15 mL of neurobasal media supplemented with 25 µM of glutamine. The result was filtered and plated. Cells were plated in 24 well plates at 10^5^ cells/well [26]. The concentration used of each compound was: 2′-dCCPA (C**1**) 5500 nM, C**2** 10 nM, CK 20 ng/mL, SCH58261 50 nM, and DPCPX 100 nM.

#### 2.2.3. Immunofluorescence

A_1_AR presence was checked using the A_1_AR polyclonal antibody ALEXA FLUOR^®^488 Conjugated, while A_2A_AR was studied by A_2A_AR antibody ALEXA FLUOR^®^ 594 Conjugated. Briefly, media was aspired, and cells fixed with fixative solution (Image-iT™ Fixative Solution, 4% formaldehyde, methanol-free, Invitrogen) for 15 min. After that, they were washed 3 times with PBS and permeabilized with a permeabilization solution (0.5% Triton X-100 solution) during 15 min. They were washed again with PBS and cell culture was incubated for 1 h with blocking buffer (Bovine Albumin Fraction V, Thermo Fisher Monza, Italy). Finally, it was proceeded with the antibody labelling (diluted 1:100). Images were acquired by optical inspection using an Olympus microscope (Olympus, Hamburg, Germany).

#### 2.2.4. Cell Treatment

**Scheme 1:**
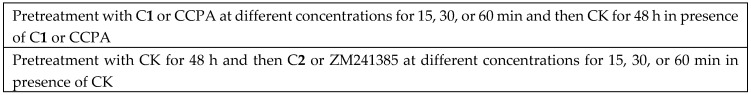
Drug application protocol.

Mixed glial cell cultures were divided into 2 groups, one to study A_1_AR partial agonists and another for A_2A_AR antagonists. Cells from the first group were pre-treated with 2 µL at three different concentrations of C**1** for 15, 30 or 60 min and then with further addition of a pro-inflammatory cocktail of cytokines (TNF-α, 20 ng/mL; IL-1β, 20 ng/mL; IFN-γ 20 ng/mL) for 48 h. The A_1_AR agonist CCPA was used as reference compound. To study C2 cells were pre-treated with the same CK cocktail for 48 h and then with 2 µL of C**2** at three different concentrations for 15, 30, or 60 min (Scheme 1). ZM241385, a well known A_2A_AR antagonist, was used as reference compound. Furthermore, ligands and CK were tested alone for 15, 30 or 60 min and 48 h respectively.

#### 2.2.5. Proliferation Assay 

1.7 × 10^4^ mixed glial cells were cultured in 98 μL of the specific medium in a 96 well plate overnight. The next day 2 μL of ligand or CK cocktail were added to the well. After all treatments, 20 µL of CellTiter 96^®^ AQueous One Solution Reagent was added in a final volume of 100 µL. Absorbance was read at 490 nm using the plate reader GENiosPro. Cell viability was calculated as percentage using the following formula:

$$\mathrm{Cell}\;\mathrm{viability}\;=\frac{\mathrm{OD}\;\mathrm{mean}\;\mathrm{of}\;\mathrm{treated}\;\mathrm{cells}}{\mathrm{OD}\;\mathrm{mean}\;\mathrm{of}\;\mathrm{control}\;\mathrm{cells}}\;\times \;100$$

An untreated control, a positive control with referent compounds and a control with water were also performed. All experiments were done in 3–5 replicates [27].

#### 2.2.6. Griess Assay

To test the antioxidant capacity of these new molecules Griess assay was performed. The Griess Reagent System is based in the conversion of sulphanilamide into an azo-compound with the presence of N-1-napthylenediamide dihydrochloride (NED) under acidic condition. 1.7 × 10^4^ mixed glial cells were cultured in 98 μL of the specific medium in a 96 well plate overnight. After 24 h the media from each well of the cell culture was transferred into a 96 well plate and then 50 µL of the sulphanilamide solution was added to each well. After 5–10 min of incubation at room temperature (rt), 50 µL of the NED Solution were dispensed to all wells and they were incubated at rt for other 5–10 min. Absorbance was measured within 30 min in the plate reader GENiosPro with a filter between 520 nm and 550 nm. This experiment was performed 3–5 replicates [28]. Concentration of NO¬¬¬¬_2_¯ in each sample was determined by comparison to a Nitrite standard reference curve.

#### 2.2.7. Hoechst Assay

This assay was carried out using Hoechst 33258. 6 × 10^5^ mixed glial cells were seeded in a six well plate. After 24 h the media was eliminated, and cell cultures washed with PBS. Then, they were washed with acetic acid/methanol solution 50:50, again washed with PBS and incubated 10 min with a fixative solution. After this, cells were cleaned with distilled water and incubated light protected for 30 min at rt with Hoechst (1 µg/mL). Finally, the dye was discarded, and cells washed with water. Glycerol solution was added and cells were observed under an Olympus microscope. The cell area and circularity were measured using ImageJ as image analyzing software [29]. Area and circularity were calculated as percentage using the following formula:

(1) $$\mathrm{Area}\;\mathrm{or}\;\mathrm{circularity}\;=\frac{\mathrm{area}\;\mathrm{or}\;\mathrm{circularity}\;\mathrm{of}\;\mathrm{treated}\;\mathrm{cells}}{\mathrm{area}\;\mathrm{or}\;\mathrm{curcularity}\;\mathrm{of}\;\mathrm{control}\;\mathrm{cells}}\;\times \;100$$

#### 2.2.8. Trypan Blue Exclusion Test

6 × 10^5^ mixed glial cells were seeded in a six well plate and after 24 h incubated with C**2** 10 nM alone or in presence of CK cocktail. After 30 min at 37 °C cells were detached with Trypsin-EDTA solution and 10 µL of cells were added to 40 μL of 0.4% trypan blue solution. Cells were counted with a Neubauer chamber and the number of live cells was calculated.

### 2.3. In Vivo Assays

#### 2.3.1. Neuroinflammation Model

Animal procedures were performed in accordance with the European Community guidelines (Directive 2010/63/EU), Portuguese law on animal care (DL 113/2013), and approved by the Instituto de Medicina Molecular Internal Committee and the Portuguese Animal Ethics Committee (Direcção Geral de Veterinária). Environmental conditions were kept constant: food and water ad libitum, 21 ± 0.5 °C, 60% ± 10% relative humidity, 12 h light/dark cycles, 3–5 rats per double-decker ventilated cage. Male Sprague Dawley rats 8 weeks old were divided into the following groups (Table 1):

C**1** was dissolved in 5% DMSO + 95% H_2_O to a final concentration of 7 mg/mL and C**2** was dissolved in DMSO to a final concentration of 5 mg/mL. Accordingly, vehicle for G1 was 5% DMSO + 95% H_2_O and for G2 was DMSO.

#### 2.3.2. Surgery

Animals were anesthetized with a cocktail of 75 mg/kg ketamine + 1 mg/kg dexmedetomidine. Once rats were deeply anesthetized, the head was fixed into a stereotaxic frame and an incision made on the scalp to expose the skull. Coordinates for injection were AP -1.0, ML ± 1.5, DV -3.7 mm. For G1, LPS was delivered bilaterally 5 µL of a 1 µg/µL LPS solution per hemisphere, and C**1** was delivered also i.c.v. 15 min before the LPS injection, 5 µL of a 7 µg/µL solution. Flow rate was kept at 1 µL/min, using an automatic injector coupled to a 10 µL hamilton syringe gauge 30. After each injection, 5 min were allowed for diffusion of the drugs before slowly removing the needle. After the procedure, rats were sutured, administered atipamezole (1 mg/kg) to reverse anesthesia, and brought back to a heated cage until fully recovered. For G2, rats were injected i.p. with a 2.5 mg/kg solution of C**2**, 30 min after the i.c.v. injection of LPS 5 µg/5 µL per hemisphere, in total of 10 µg. Rats were monitored for sickness behavior (body temperature, general activity) and given access to gel food in the first 48 h.

#### 2.3.3. Open Field

Two days after the neuroinflammatory insult, Open Field (OF) was performed. This experiment uses the natural exploratory behavior of the animal when placed in a novel environment to measure general locomotor activity and exploratory behavior. It was used to determine whether normal exploratory activity was recovered at the time of the behavior tests, to avoid possible confounds due to sickness behaviors during memory test performance. Animals were placed in an arena (40 × 40 × 40) for 5 min in a dim-lighted room and activity monitored using smart video tracking software. Parameters measured included distance covered, velocity, and permanence time in periphery vs. center area of the arena [30].

#### 2.3.4. Y Maze

Y Maze is a behavior test useful to measure short-term spatial working memory. Animals are introduced in the maze always in the same starting position, at the end of the start arm. An initial habituation phase of 8 min, animals freely explore 2 of the arms, the 3^rd^ being closed. Sixty minutes after the habituation period animals are placed again in the maze for 5 min this time with all 3 arms available to exploration. Using the Smart video tracing software, each animal’s route was registered [31]. The time spent in the arm initially closed, which for the animal is the novel, unexplored environment, is calculated in relation to the time spent in the already familiar arms. 

### 2.4. Statistical Analysis

Results were represented as mean of 3–5 replicates ± standard error (±SE) for all experiments. Biological data were analysed using Prism 5.0 programme (GraphPAD Software, San Diego, CA, USA). Statistical analysis was performed using one-way ANOVA or two-way ANOVA in the case of Y Maze and Open Field. A *p*-value < 0.05 was considered to indicate a significant difference.

## 3. Results

### 3.1. In Vitro Studies in Mixed Glial and Neuronal Cells 

**Figure 2:**
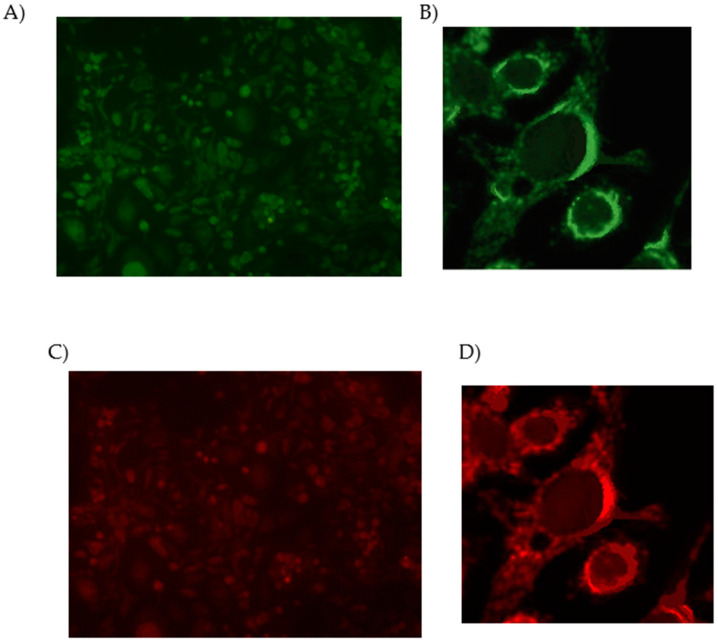
Double immunofluorescence staining in mixed glial cell cultures. (**A**) Cells were treated with Adenosine A_1_ Receptor Polyclonal Antibody ALEXA FLUOR^®^ 488 Conjugated (green). (**B**) Higher magnification displaying A_1_AR expression on the plasma membrane. (**C**) Adenosine A_2A_ Receptor antibody ALEXA FLUOR^®^ 594 Conjugated (red). (**D**) Higher magnification displaying A_2A_AR expression on the plasma membrane.

Primary mixed glial and neuronal cells obtained from new-born rat pups or embryos, respectively, were chosen as powerful tools for studying A_1_AR and A_2A_AR involvement in mechanisms underlying microglial inflammatory responses in the central nervous system (CNS). Initially, it was evaluated the presence of these receptors in both cell cultures through direct immunocytochemistry using the A_1_AR polyclonal antibody ALEXA FLUOR^®^ 488 and A_2A_R antibody ALEXA FLUOR^®^ 594 Conjugated [32]. Results showed that both receptors are present in suitable amounts to undertake the pharmacological experiments with A_1_AR and A_2A_AR ligands (Figure 2).

After confirmation that the cellular models express both receptors, ligand effects on cell viability it was assessed. Firstly, the CellTiter 96^®^ AQueous One Solution Cell Proliferation assay was performed. Each ligand was tested at three different concentrations according to the K_i_ values obtained in binding assays. Specifically, we used a lower, a similar, and a higher concentration than the K_i_ value. The concentrations used for A_1_AR partial agonist 2′-dCCPA (C**1**) were 2, 5.5, and 30 µM while for A_2A_AR antagonist (C**2**) were 5, 10, and 60 nM at 15, 30, and 60 min of incubation in comparison with reference compounds CCPA and ZM241385, respectively. Since the K_i_ values of CCPA for A_1_AR and ZM241385 for A_2A_AR (K_i_ = 1.2 nM) are the same, in cell viability experiments we used the concentrations 5, 10, and 60 nM at 15, 30, and 60 min. 

**Figure 3:**
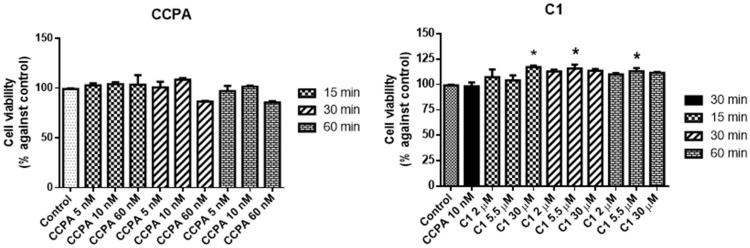
Effects of 2-chloro-N^6^-cyclopentyladenosine (CCPA) and C**1** on mixed glial cell culture viability. Percentage of cell viability after treatment with different ligand concentrations (2–30 μM) for 15, 30, or 60 min of incubation. Results represent the average of 3–5 independent experiments. * *p* < 0.05 of treated cells against control.

The highest effect for both CCPA and ZM241385 was observed for 10 nM after 30 min of incubation (Figure 3).

Results comparing CCPA and C**1** are shown in Figure 3.

At 15 min of C**1** incubation, only the highest tested 30 µM concentration produced a significant increase in cell viability (117 ± 1.3 vs. control) whereas for 30 min already at 5.5 µM the effect was visible (116 ± 1.7 vs. control). The effects measured after 30 min are similar to those obtained after 60 min of incubation. CCPA alone did not change cell viability significantly.

The A_2A_AR antagonist, C**2**, was tested in comparison with ZM241385.

**Figure 4:**
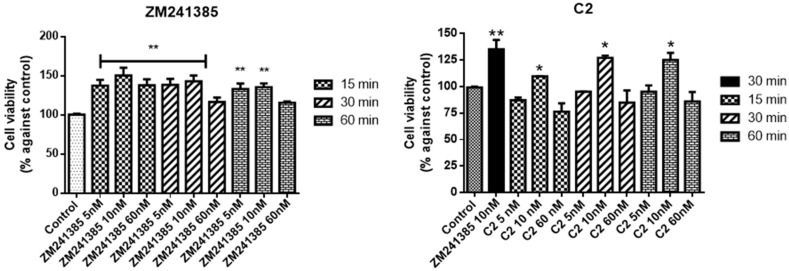
Effects of ZM241385 and C**2** on mixed glial cell culture viability. Percentage of cell viability after treatment with the ligand after 15, 30, or and 60 min of incubation. Results represent the average of 3–5 independent experiments. * *p* < 0.05, ** *p* < 0.01 of treated cells against control.

When compared to control, C**2** increases cell viability at 10 nM at all incubation times (Figure 4, 110 ± 2.7, 127 ± 3.1, and 125 ± 4.3 vs. control) despite not reaching the reference compound ZM241385 at the same concentration (135 ± 3.5 vs. control). In addition, at the highest concentration of C**2**, 60 nM, the cell viability is lower than the control suggesting a tendency to toxicity (values nearly 80%).

It was then tested whether these compounds could provide protection in in vitro inflammatory model. For this, mixed glial cell cultures were exposed to a pro-inflammatory cocktail of CK constituted by TNF-α, IL-1β, and IFN-γ 20 ng/mL for 48 h. As expected, this inflammatory insult decreased cell viability (70 ± 3 vs. control).

**Figure 5:**
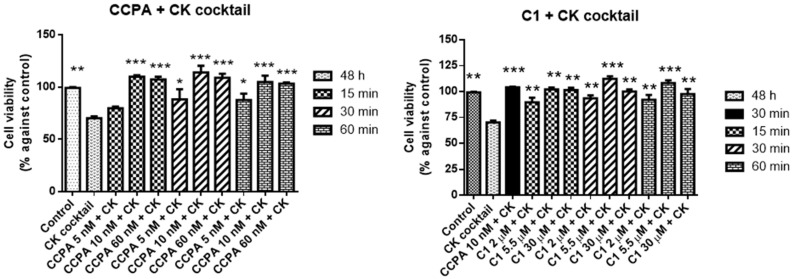
Protective effects of CCPA and C**1** against CK aggression on mixed glial cell culture. Percentage of cell viability after treatment with the CK cocktail for 48 h. Results represent the average of 3–5 independent experiments. * *p* < 0.05, ** *p* < 0.01, *** *p* < 0.001 of treated cells against CK.

The neuroprotective effect of the compounds against a neuroinflammatory insult was studied performing different experiments. In the first experiment, cells were pre-treated with C**1** at 2, 5.5, and 30 µM for 15, 30, and 60 min before adding the CK cocktail for 48 h. CCPA, 10 nM at 30 min, that showed the best effect, was used as reference. C**1** and CCPA were present for all experiment duration. Results are reported in Figure 5.

C**1** is able to prevent the CK aggression as well as CCPA reporting the cell viability values to the control or slightly higher. The highest effect was observed at 5.5 µM for 30 min (113 ± 3.1 vs. control).

**Figure 6:**
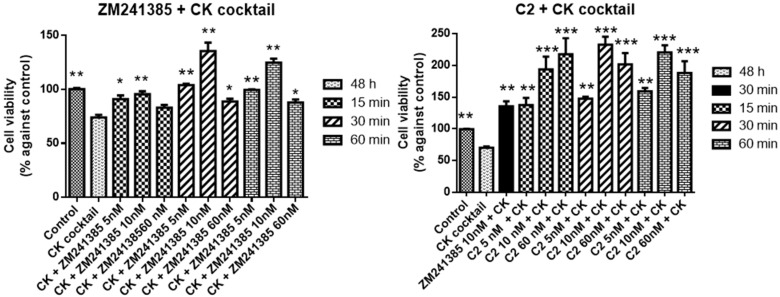
Restoring effects of ZM241385 and C**2** against CK aggression on mixed glial cell culture. Results represent the average of 3–5 independent experiments. * *p* < 0.05, ** *p* < 0.01, *** *p* < 0.001 of treated cells against CK.

In the second experiment cells were pretreated with 20 ng/mL of CK cocktail for 48 h and then the C**2** at 5, 10, and 60 nM for 15, 30, and 60 min was added or the referent compound ZM241385 at the same concentration and incubation time. Results were reported in Figure 6.

C**2** was able to rescue the inflammatory damage caused by CK exposure, already at 5 nM for 15 min (138 ± 1.7 vs. control) and the rescue was comparable to the reference compound ZM241385 at 10 nM for 30 min (136 ± 1.7 vs. control). The highest increase was shown at 10 nM for 30 min (233 ± 3.4 vs. control). 

Since the best incubation time was 30 min, in the next set of experiments, neuronal cells were treated with the dose that showed the best effect of C**1** (5.5 µM), C**2** (10 nM), CCPA (10 nM), and ZM241385 (10 nM) alone or in combination with CK. 

**Figure 7:**
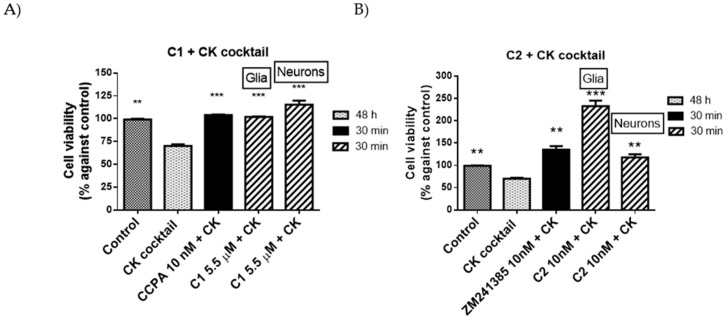
Protective (**A**) and restoring (**B**) effects reference compounds and compounds **1** and **2** against CK aggression on mixed glial cell culture and neurons. Percentage of cell viability after treatments. Control, CK, and reference compound results were obtained in glial cell culture. Results represent the average of 3–5 independent experiments. ** *p* < 0.01, *** *p* < 0.001 of treated cells against CK.

Similar results to those obtained in mixed glial cells were obtained in neuronal cells and are reported in Figure 7.

**Figure 8:**
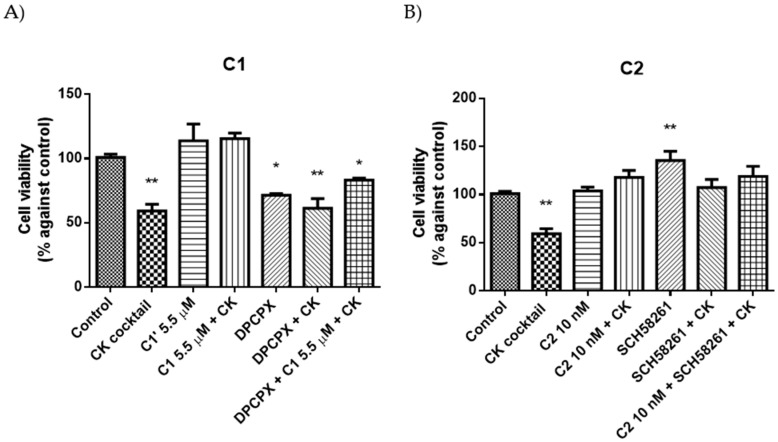
Protective (**A**) and restoring (**B**) effects of C**1** and C**2**, respectively, in presence or absence of A_1_AR (DPCPX) or A_2A_AR (SCH58261) antagonists in presence or in absence of CK. Percentage of cell viability after treatments. Results represent the average of 3–5 independent experiments. * *p* < 0.05, ** *p* < 0.01 of treated cells against control.

In addition, in neurons parallel experiments were performed with selective A_1_AR antagonist DPCPX (K_i_A_1_AR = 3.9 nM) and an A_2A_AR antagonist SCH58261 (K_i_A_2A_AR = 1.2 nM) in order to verify the involvement of these receptors in obtained results. Concentrations used of DPCPX and SCH58261 were 100 nM and 50 nM, respectively. Results showed that C**1** is able to partially counteract the effect of DPCPX in presence of CK. Moreover, the combination of the two antagonists C**2** and SCH58261 did not exhibited a synergic effect (Figure 8).

In summary, it was demonstrated that C**1** and C**2** have beneficial effects in protecting neurons against neuroinflammatory insults interacting with A_1_AR and A_2A_AR, respectively. Moreover, C**2**, was neuroprotective when administered after the inflammatory stimulus to a larger degree than the reference compound.

**Figure 9:**
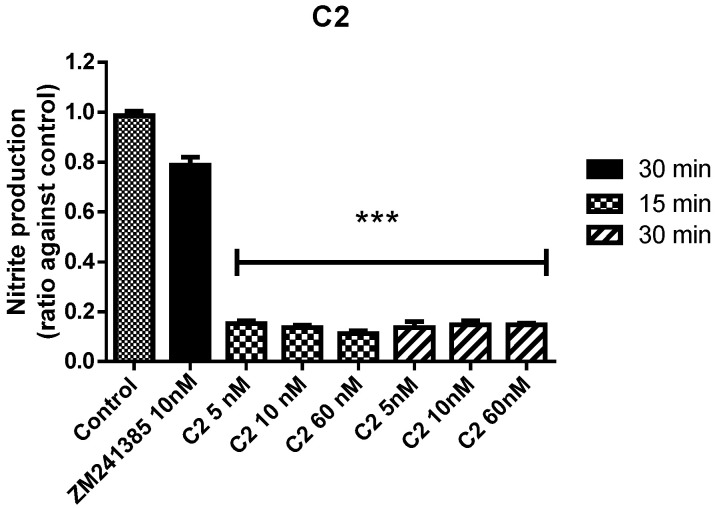
Antioxidant effects of ZM241385 and C**2** on mixed glial cell culture. Ratio of NO¬¬¬¬_2_¯ production after treatments. Results represent the average of 3–5 independent experiments. *** *p* < 0.001 of treated cells against control.

Under normal conditions in the brain, in order to preserve cell functions, the immunological responses counteract the formation of reactive oxygen (ROS) and nitrogen (RNS) species. Since high levels of ROS and RNS species are present in neuroinflammation and associated pathologies, the antioxidant profile of the compounds were evaluated. Compounds **1** and **2** were tested, through Griess Reagent System, using the same conditions as for the precedent experiments. Specifically, C**1** (5.5 µM), C**2** (10 nM), CCPA (10 nM), and ZM241385 (10 nM) were incubated for 30 min. Compounds were tested alone and in combination with CK in comparison with reference compounds. C**1** did not show any antioxidant activity (data not shown). On the contrary, C**2** displayed an important antioxidant ability. The levels of nitrite produced, after C**2** treatment, were lower than those obtained in control, demonstrating that this compound possess a strong antioxidant activity (Figure 9). 

**Figure 10:**
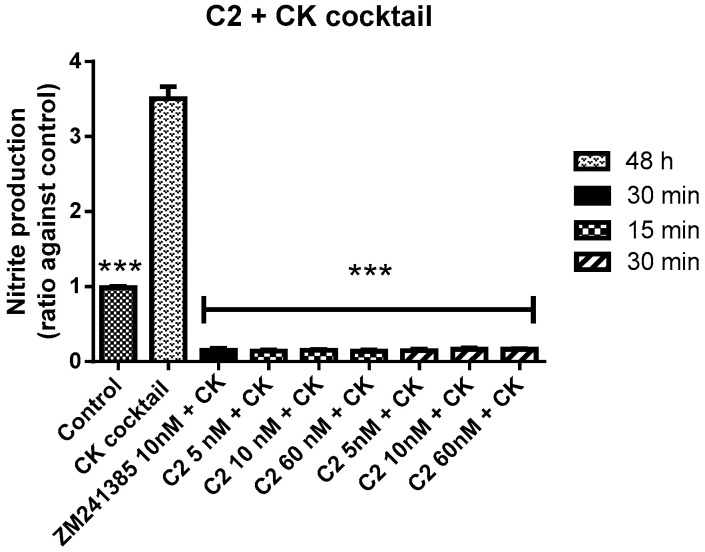
Antioxidant effects of ZM241385 and C**2** after pre-treatment with CK cocktail on mixed glial cell culture. Ratio of NO¬¬¬¬_2_¯ production after treatments. Results represent the average of 3–5 independent experiments. *** *p* < 0.001 of treated cells against CK.

In presence of CK cocktail, C**2** was able to decrease the high nitrite levels produced by the CK cocktail or control (Figure 9). It is important to note that, both C**2** and the reference compound were in these conditions able to reduce the oxidative load induced by the inflammatory stimulus, CK aggression (Figure 10).

**Figure 11:**
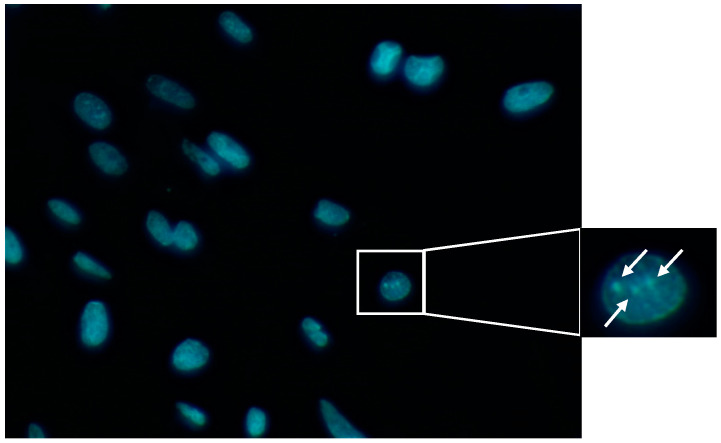
Example of an image obtained through Hoechst assay. In the picture is pointed the chromatin condensation.

TNF-α and IFN-γ have been shown to synergistically induce apoptosis via the induction of nitric oxide [33]. Also, IL-1β can induce cell death by apoptosis [34]. Generally, anti-apoptotic properties that lead to a great neuron survival involve reduction in apoptosis mediators as well as oxidative substances, such as superoxide dismutase and hydrogen peroxide. In order to study the CK cocktail effect in cell morphology the Hoechest assay was performed in mixed glial cells. Image analysis revealed a significant decrease in cell circularity and area detected in cells treated with CK compared to the control cells (*p*-values < 0.05) (Figure 11). 

**Figure 12:**
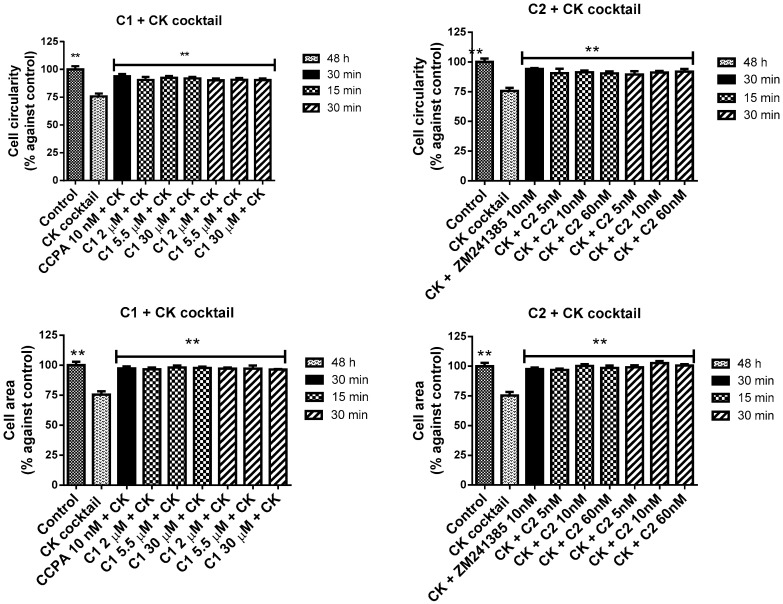
Cell area and circularity after the pre and post treatment with CCPA or C**1** and ZM241385 or C**2** respectively in presence of the CK cocktail in mixed glial cell cultures. The results represent the average of 3–5 independent experiments. ** *p* < 0.01 of treated cells against CK.

Results correlate positively and significantly with those obtained by cell viability assay (R = 0.988, *p*-value = 0.049). Effects obtained with compounds **1** and **2** in pre and post treatment, respectively, indicated that they have a protective effect and contrast the CK aggression in a statistically significant way (Figure 12).

Since these compounds showed good biological effects at the cell level, the study continued on in vivo rat neuroinflammation model in order to test their in vivo potential.

### 3.2. In Vivo Studies Rat Models

Compounds **1** and **2** were tested in an in vivo neuroinflammatory rat model. To induce a neuroinflammatory insult, rats were administered intracerebroventricularly lipopolysaccharide (LPS, 8–10 ug). Treatment groups were divided into LPS + saline (*n* = 8), LPS + C**1** (*n* = 8), LPS + C**2** (*n* = 8), Vehicle + C**1** (*n* = 5), Vehicle + C**2** (*n* = 5), Vehicle + saline (*n* = 5). Behaviour tests were performed 3 days post-insult, after sickness behaviour had subsided. 

#### 3.2.1. Open Field

**Figure 13:**
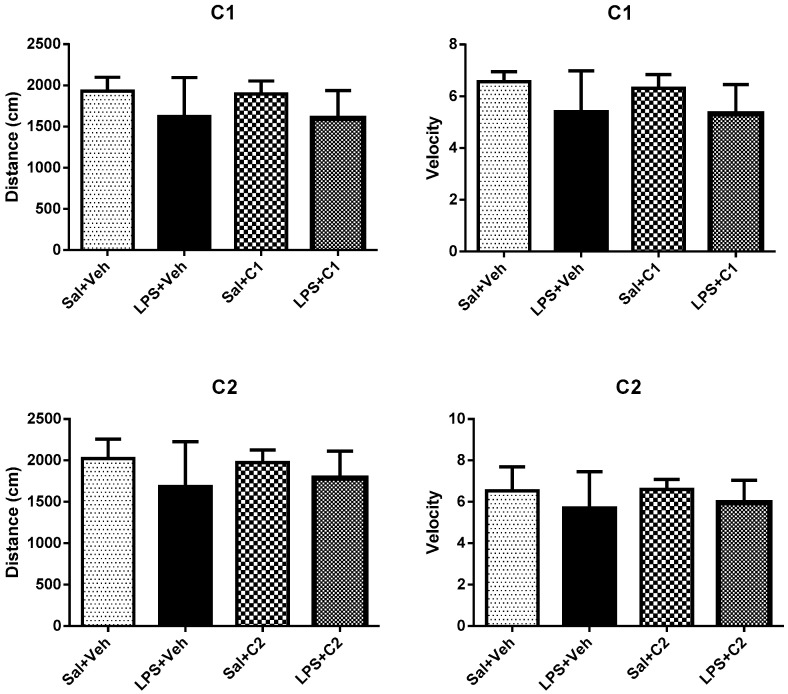
Effects of treatment in distance and velocity performed by rats in Open Field. Saline (sal), Vehicle (veh, vehicle used for the ligand dilution).

Rats were placed in an open arena and observed for 5 min. Their general behavior was evaluated and both velocity and distance travelled measured. No significant differences were observed between the various groups (Figure 13).

**Figure 14:**
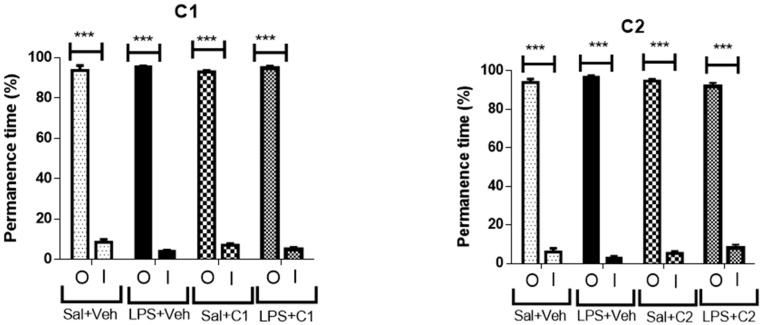
Effects of treatment in permanence time in the outside area (O) and inside area (I) in Open Field. Saline (sal), Vehicle (veh, vehicle used for the ligand dilution). *** *p* < 0.001 of inside area against outside area. Inside areas did not demonstrate to be statistically different among them.

The percentage of time animals spent in the peripheral vs. central areas in the arena was also calculated. Naturally, rodents feel safer near the walls (periphery), since these provide safety from any predators, but also have a biological drive to explore new environments, and should spend some time in the arena centre. All groups, independently of the treatment or insult, spent similar amounts of time between the periphery and the centre area, suggesting that no changes in anxiety-like behaviours results from any of the insult/treatment pairing (Figure 14).

#### 3.2.2. Y Maze

**Figure 15:**
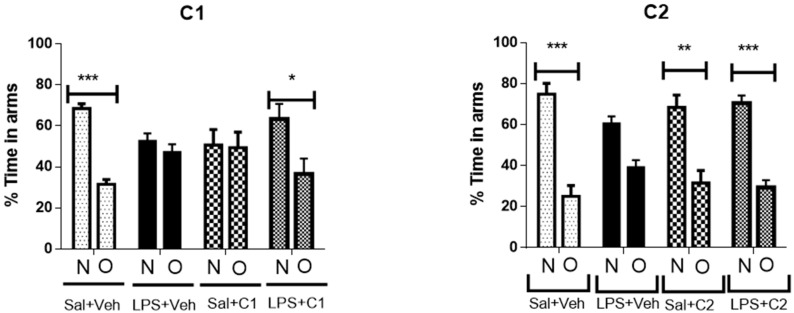
Effects of treatment in time spent in novel (N) and old (O) arms. Saline (sal), Vehicle (veh, vehicle used for the ligand dilution). ** *p* < 0.05, ** *p* < 0.01, *** *p* < 0.001 of novel area against old area.

After assessing that locomotor behaviour was preserved and not different between the groups it was evaluated short-term memory using the Y Maze. In this paradigm, animals are left to explore the maze with one of the Y maze arms closed off, for 8 min. After 1 h the animal is reinserted in the maze, but this time all arms are available for exploration. The animal’s natural behaviour is to spend more time exploring the previously closed off arm, since this is a new environment, and animals have a natural preference for novelty. The arms that were accessible from the beginning are considered the “other arms” (O), while the “new unexplored arm” is the novel arm (N). Control groups (Vehicle + Saline), as expected, showed a preference for the novel arm (Figure 15). Upon a neuroinflammatory stimulus with LPS, animals showed no preference for the novel arm, as can been seen in the LPS + saline groups, demonstrating that this insult produces impairment in short-term memory performance. This phenotype was rescued by administration of either C**1** or C**2** compounds, suggesting that these compounds are able to neuroprotect cells from the effects of the inflammatory insult. When administered alone, C**1** resulted in a reduction in the short-term memory performance, while C**2** was devoid of any effects in memory performance.

## 4. Discussion

Neuroinflammation is a complex protective response of the brain against harmful agents, such as pathogens, toxins, and traumatic shocks or factors that induce neurodegeneration. Nevertheless, this response needs to be controlled and last for a short period; otherwise, it may to be an important factor for major neurodegenerative and psychiatric disorders. This inflammation is mediated by the production of CK, chemokines, reactive oxygen species, and secondary messengers. 

The most common neuropathology in almost all CNS diseases is characterized in changes in microglial morphology. In fact, microglia are considered the immune cells in the CNS and their activation is a significant common cause of neuropathology in CNS diseases [35]. These immune cells express ARs where adenosine plays an important immunoregulatory role. The effects on the inflammation regulation by adenosine suggest that the use of selective agonists or antagonists able to activate or inactivate ARs could have important therapeutic implications in several diseases. Results obtained with immunocytochemistry demonstrated the presence of A_1_AR and A_2A_AR in mixed glial and neuronal cells. This work is designed to study the stimulation of A_1_AR and the block of A_2A_AR in in vitro and in in vivo models of neuroinflammation. The limitations of full AR agonists as well as A_1_AR agonist for human therapies are correlated to numerous side effects and agonist-induced desensitization of receptor [36]. In order to overcome these drawbacks, the partial agonist 2′-dCCPA (C**1**) was chosen to study the effect in neuroinflammation. In addition, the new C**2**, belonging to the series of 8-substituted 9-ethyl-2-phenethoxyadenines synthesized in house, proven to be a potent antagonist with a subnanomolar affinity versus A_2A_AR (K_i_ = 0.75 nM, IC_50_A_2A_AR = 251 nM).

Some evidence suggests that the reduction of A_1_AR in microglia, macrophage, and neurons leads to state of neuroinflammation that occurs by enhancement of pro-inflammatory response and the release of CK [37,38]. On this base, the experiments with A_1_AR partial agonist C**1**, which produces A_1_AR activation, were performed using the compound before the aggression by CK cocktail determining the neuroprotective effect against CK insult. On the other hand, the overexpression of A_2A_AR associated to aging and chronic stress, combined with evidence that glial A_2A_AR participates in neurodegeneration induced by A_2A_AR stimulation [30,39], directed the experiments towards an administration of A_2A_AR antagonist C**2** after the aggression by CK cocktail.

Mixed glial and neuronal cells responses were examined after pro-inflammatory stimuli by a CK cocktail (TNF-α, IL-1β, and IFN-γ) and in presence of the A_1_AR partial agonist C**1** and A_2A_AR antagonist C**2**. The choice of these CK was supported by several line of evidence. In many studies, as a pro-inflammatory stimulus, IFN-γ and TNF-α were used in an in vitro neuroinflammation model since they bind to receptors on microglia and other brain cells [40,41,42,43]. In addition, the use of IL-1β for microglia activation is usually related to neuroinflammation. In neurodegenerative disorder, the level of all these CK are elevated and this chronic high level is implicated in initiating and/or maintaining glial activation in an in vivo experimental model of Parkinson’s disease [44,45]. Experiments were performed with compounds **1** and **2** in comparison with reference compounds. The agonist CCPA was used as reference compound for A_1_ARs. The antagonist ZM241385 was in turn used as a reference compound of A_2A_AR since it is able to reduce activation of microglia and to downregulate pro-inflammatory CK expression [46]. It is important to note that compounds **1** and **2** exhibited in vitro good cell viability like reference compounds CCPA and ZM241385, showing a no toxic effect. In addition, a pilot experiment was performed up to 72 h in order to verify if these compounds were toxic after a long exposition. Results showed that only a not significant decrease (*p* < 0.05) of cell viability was observed after this long time of exposition.

The pretreatment of cells with C**1**, at 5.5 µM for 30 min of incubation, prevents completely the CK aggression as well as CCPA. 

In addition, C**2** increased cell viability after the inflammatory lesion in a concentration-dependent manner. The highest effect of C**2**, at 10 nM for 30 min of incubation, was of 233 ± 3.4 vs. control (Figure 6). This might suggest a pathological proliferation effect of glial cells after the inflammatory insult with morphological changes, increase of ROS and RNS levels, but when glial cells were treated with C**2** alone did not increase the RNS levels. In addition, when glial cells were pre-treated with CK followed by C**2**, the RNS levels were lower than control, underlining the improbability of a pathological proliferation. Moreover, experiments with trypan blue stain were performed in order to consolidate this hypothesis and understand if results were due to an increase of metabolic processes or to an enlarged number of cells. Results obtained in presence of C**2** alone at 10 nM confirmed a high viability over 30 min of incubation (123 ± 4.5 vs. control). Results reported in presence of C**2** after the aggression with CK cocktail showed that the number of cells was higher than control, underlining that C**2** has a surviving effect (129 ± 3.5 vs. control). Comparing this result with that obtained with the MTS assay of C**2** (233 ± 3.4 vs. control), it can be hypothesized that the high viability is explained both by an increased cell proliferation and by an increase in cellular metabolic processes. Hence, C**2**, at 10 nM for 30 min of incubation, has potential to rescue the inflammatory damage more than reference compound ZM241385. 

The involvement of A_1_AR and an A_2A_AR, in obtained results, was demonstrated by the use of A_1_AR antagonist DPCPX (K_i_A_1_AR = 3.9 nM) and an A_2A_AR antagonist SCH58261 (K_i_A_2A_AR = 1.2 nM) (Figure 7). A partial agonist C**1** in fact, counteracts only partially the effect of DPCPX in presence of CK due of its intrinsic activity of 0.70. On the other hand, the combination of C**2** with SCH58261 did not exhibited a synergic effect (Figure 7). This result is explained by the different concentrations used of these antagonists since SCH58261 (50 nM) is 5 folds higher than C**2** (10 nM) but their A_2A_AR affinity are similar (SCH58261 K_i_A_2A_AR = 1.2 nM and C**2** K_i_A_2A_AR = 0.75 nM). In these conditions, receptors were mainly bound by SCH58261 and the addition of C**2** is unable to produce a synergic effect.

In the CNS redox activity is essential for cell metabolic processes and functions. To preserve cell functions in the brain, the normal formation of reactive oxygen and nitrogen species is controlled by immunological responses. On the other hand, in pathological conditions, it was demonstrated a cross-talk between pro-inflammatory and oxidative signals that can lead to neuronal damage and subsequent neurodegeneration [47]. In addition, mitochondrial ROS induces the activation of mitochondrial apoptotic proteins, leading to cellular apoptosis and organ damage. 

The C**2** demonstrated that is able to decrease NO¬¬¬¬_2_¯ levels induced by CK more than the well know ZM241385, showing a potential benefit in neuroinflammation.

Neurodegenerative diseases are characterized by neuronal death and progressive neuroinflammation. The exact mechanisms of neuronal death remain elusive but seems that the programmed cell death (PCD) apoptosis plays a critical role [48]. Neuroinflammation and glial activation are implicated in apoptosis process, since compounds **1** and **2** showed a cell protective effect reducing the aggression of pro-inflammatory CK cocktail in vitro it is possible to hypothesize that they possess an antiapoptotic effect.

To test the in vivo efficacy of these compounds, it was chosen a neuroinflammatory model using LPS delivered directly into the CNS, which it enabled to circumvent the peripheral effects of systemic inflammation. Nonetheless, in the first 24 h post-surgery animals displayed the characteristic sickness behavior with increased body temperature, and lethargy that subsided after 48 h. In fact, when animals were tested in the open field arena post 48 h, locomotor activity, was indistinguishable between control and LPS insult. However, when tested for short-term memory the LPS group presented impaired performance, indicating that neuroinflammation had resulted in altered brain function. Both pre-treatment with the A_1_AR agonist C**1** and post-treatment with the A_2A_AR antagonist C**2** were efficient in rescuing the short-term memory performance, suggesting that these compounds can provide neuroprotection, presumably by a reduction in reactive oxygen species as seen in the cell assays.

The effect variability of LPS alone (Figure 15) between experimental G1 and G2 could be explained by the body weight difference of these two animal group, which in G1 animals had 7 weeks of age and weighed between 200 and 300 g. In the second group all animals had 8 weeks and weighed around 300 g. Despite having adjusted the LPS dose slightly for body weight to account for the differences, it is possible that the more pronounced effect in behavior was a reflex of this higher total LPS dose in the first group. Nonetheless, G2 also presented impaired short-term memory performance.

C**1** alone also induced a slight impairment in short-term memory performance. It is well known that A_1_AR agonists produce a marked depression of cellular activity and a generalized inhibition of cellular function, in fact acute i.p. administration of A_1_AR agonists produces marked effects on locomotor activity, dependent of CNS-mediate effects [23]. This is in accordance with A_1_AR activation that produces, in the absence of an insult, deleterious consequences, at least when directly injected into the CNS. This general inhibitory role of cellular functions is one of the best known effects of adenosine, in situations of damage to the CNS, acting as an energetic sensor that promotes marked inhibition of all synaptic activity. It is also well established that preconditioning tissues with an exposure to an A_1_ agonist, provides neuroprotection to a subsequent insult or damage, and the outcome is improved cellular function and behavior [49]. Accordingly, blockade of A_2A_AR is more relevant to stop the deleterious effects of A_2A_AR over activation in these situations.

Since C**1** was delivered directly into the brain, we are confident it acted in the affected brain areas, while C**2**, administered by i.p. injection was also able to provide rescue from the CNS inflammatory insult, suggesting it readily crosses the BBB.

## 5. Conclusions

Pro-inflammatory CK cocktail promotes neuroinflammation and cellular apoptosis, which can be alleviated by compounds **1** and **2**. C**1** was able to prevent the inflammatory effect induced by CK while C**2** has both anti-inflammatory and antioxidant properties, preventing both neuroinflammation in mixed glial cells and in an animal model of neuroinflammation. This suggests C**2** as a potential candidate for neuroinflammation therapy. Collectively, our data provide novel evidence to use potent and selective A_1_AR partial agonists and A_2A_AR antagonists as a promising therapeutic approach to improve the functionality of patients with problems associated with an oxidative stress and age-associated disease.

## Figures and Tables

**Table 1:** Experimental groups 1 (G1) and 2 (G2): LPS (lipopolysaccharide) was delivered intracerebroventricularly (i.c.v.) using stereotaxic coordinates, 10 ug total was injected, unless otherwise noted. C**1** was delivered i.c.v., while C**2** was delivered intraperitonially (i.p.). Control groups included i.c.v. saline delivery and vehicle, either i.c.v. or i.p., depending on compound delivery route.

	Drugs (Delivery Route)	LPS (icv)	Saline (icv)
G1	Compound **1** (icv)	LPS + C**1** (*n* = 8)	Saline + C**1** (*n* = 5)
	Vehicle (icv)	LPS + Veh (*n* = 8)	Saline + Veh (*n* = 5)
G2	Compound **2** (i.p.)	LPS + C**2** (*n* = 8)	Saline + C**2**(*n* = 5)
	Vehicle (ip)	LPS + Veh (*n* = 8)	Saline + Veh (*n* = 5)
